# Search Strategy Analysis of 5xFAD Alzheimer Mice in the Morris Water Maze Reveals Sex- and Age-Specific Spatial Navigation Deficits

**DOI:** 10.3390/biomedicines11020599

**Published:** 2023-02-17

**Authors:** Carolina Quintanilla Sánchez, Franziska W. Schmitt, Nadine Curdt, Anna Celine Westhoff, Irina Wanda Helene Bänfer, Thomas A. Bayer, Yvonne Bouter

**Affiliations:** 1 Department of Psychiatry and Psychotherapy, Division of Molecular Psychiatry, University Medical Center (UMG), Georg-August-University, 37075 Goettingen, Germany; 2 Department of Nuclear Medicine, University Medical Center Göttingen (UMG), 37075 Goettingen, Germany

**Keywords:** cognitive impairments, mouse model, Alzheimer’s disease (AD), search strategy analysis, navigation strategy, 5xFAD mice, sex differences, spatial reference memory

## Abstract

Spatial disorientation and navigational impairments are not only some of the first memory deficits in Alzheimer’s disease, but are also very disease-specific. In rodents, the Morris Water Maze is used to investigate spatial navigation and memory. Here, we examined the spatial memory in the commonly used 5xFAD Alzheimer mouse model in a sex- and age-dependent manner. Our findings show first spatial learning deficits in 7-month-old female 5xFAD and 12-month-old male 5xFAD mice, respectively. While the assessment of spatial working memory using escape latencies provides a global picture of memory performance, it does not explain how an animal solves a spatial task. Therefore, a detailed analysis of swimming strategies was performed to better understand the behavioral differences between 5xFAD and WT mice. 5xFAD mice used a qualitatively and quantitatively different search strategy pattern than wildtype animals that used more non-spatial strategies and showed allocentric-specific memory deficits. Furthermore, a detailed analysis of swimming strategies revealed allocentric memory deficits in the probe trial in female 3-month-old and male 7-month-old 5xFAD animals before the onset of severe reference memory deficits. Overall, we could demonstrate that spatial navigation deficits in 5xFAD mice are age- and sex-dependent, with female mice being more severely affected. In addition, the implementation of a search strategy classification system allowed an earlier detection of behavioral differences and therefore could be a powerful tool for preclinical drug testing in the 5xFAD model.

## 1. Introduction

Alzheimer’s disease (AD) is the most common neurodegenerative cause of dementia, representing 60–80% of cases [1]. AD is characterized by the accumulation of β-amyloid (Aβ) plaques, phosphorylation of tau protein, chronic inflammation with micro- and astrogliosis, and neuron loss [1,2]. Theses abnormalities can be detected as early as 20 years before the onset of symptoms, and usually begin in the hippocampus and progressively spread to other brain regions. It is noteworthy that women are more frequently and severely affected by the disease than men. However, there is still no clear explanation for this discrepancy [3,4,5,6,7].

Spatial disorientation and navigation impairments are not only some of the earliest symptoms of memory deficits in AD patients, but are also very specific for the disease [8,9]. Patients in the early stages of symptomatic AD as well as patients with mild cognitive impairment (MCI) often have difficulties to orientate themselves in unfamiliar and even familiar places [10,11]. There are two main types of strategies used for spatial navigation: allocentric memory (or spatial reference memory), which is hippocampus-dependent, and egocentric memory, described as hippocampus-independent. Allocentric memory relies on surrounding landmarks to locate a specific goal, whereas egocentric memory depends on self-positioning and heading as a reference for orientation [12,13]. Following a spatial-temporal pattern, spatial reference memory is affected first, however, patients can often compensate these deficits with egocentric memory strategies. As AD progresses, egocentric memory is also impaired [13,14,15,16,17]. 

Spatial navigation and memory have extensively been investigated in rodents using the Morris Water Maze (MWM), a test developed by Richard Morris in 1984 [18]. The animals learn to use distal and proximal cues to locate a hidden escape platform and navigate to it in the most direct way possible. Learning in the MWM can be quantified by analyzing the latency to reach the platform, path efficiency, average platform distance, or swimming distance. In addition, immobility or swimming speed are commonly used as control parameters. Advantages of the MWM include its cross-species usability and robustness to motivational and motor factors [19]. Therefore, the test is routinely used in studies to investigate disease development and progression or to investigate the efficacy of therapeutic interventions [20,21,22,23,24,25]. However, despite the strength of the MWM, the single measurements traditionally used cannot accurately reflect the complexity of searching and swimming behavior, and do not provide a detailed picture of how an animal solves a spatial navigation task [26,27]. Therefore, the goal of the present work was to identify possible spatial navigation deficits in the MWM in the commonly used 5xFAD model by studying the search strategies.

The 5xFAD model is a widely used AD mouse model created by Oakley and colleagues in 2006 that carries five familial human AD mutations in the amyloid precursor protein (APP) and the presenilin-1 (PSEN1) gene [28]. Consequently, 5xFAD mice develop a robust and progressive plaque pathology and gliosis as well as synaptic dysfunction and neuronal loss [28,29]. Furthermore, 5xFAD mice show age-dependent memory and motor deficits [30,31,32,33,34]. 

In the present study, we investigated spatial learning and memory of 5xFAD and WT mice in a sex- and age-dependent manner. The aim of the current work was to conduct a detailed analysis of the swimming strategies in the MWM for a better understanding of the previously described memory deficits in 5xFAD animals. Therefore, the swimming strategies of male and female 5xFAD mice were analyzed in the MWM along with conventional escape latencies.

## 2. Materials and Methods

### 2.1. 5xFAD Transgenic Mice

5xFAD mice overexpress the 695 amino acid isoform of the human APP carrying the Florida (I716V), London (V717I), and Swedish (K670N/M671L) mutations under the control of the neuron-specific murine Thy1-promoter. Furthermore, human presenilin-1 (PS1), which carries the L286V and M146L mutations, is expressed under the control of the Thy-1 promoter [28]. 5xFAD mice (Jackson Laboratories, Bar Harbor, ME, USA) used in this study were kept on a C57Bl/6J genetic background [34,35], and wild type (WT) littermates served as age-matched control animals. All animals were handled according to the German guidelines for animal care and approved by the local authorities (Niedersächsisches Landesamt für Verbraucherschutz und Lebensmittelsicherheit, Röverskamp 5, 26203 Oldenburg, Germany), and all experiments followed the recommendations in the ARRIVE guidelines.

### 2.2. Morris Water Maze

Spatial reference memory was evaluated in the Morris Water Maze (MWM) as previously described [27,36,37]. 

Briefly, testing was conducted in a circular pool with a diameter of 110 cm filled with tap water and non-toxic white paint. Mice were trained to search for a hidden circular platform (10 cm) using distal and proximal cues. The water temperature was maintained at 20 ± 2 °C. The pool was divided into four virtual quadrants based on the platform localization: right, left, opposite, and target. The experimental procedures consisted of 3 phases: cued training, acquisition training, and a final probe trial. 

The cued training lasted for 3 days (4 trials per day with 15 min intertrial interval, maximum duration of 60 s) and the platform was marked with a flag. Mice were introduced into the pool facing the wall, and the platform position and introduction points changed randomly across the trials. Each mouse had 60 s to reach the platform, otherwise they were gently guided to it. Once on the platform, the mice were given 10 s before being removed from the arena. To avoid hypothermia, mice were placed in front of a heat lamp between the trials. 

The acquisition phase started 48 h after the last day of cued training and lasted 5 days (4 trials per day with 15 min intertrial interval, maximum duration of 60 s). Acquisition was conducted similarly to cued training, with the exceptions that the platform remained stationary and the flag was removed. In addition, proximal cues were attached to the edge of the pool. 

Finally, a probe trial was conducted 24 h after the last day of acquisition training to assess spatial reference memory. During a single 60 s trial, the platform was removed from the pool and mice were introduced to the pool from a novel entry point (opposite from the platform). ANY-Maze video tracking system was used to record escape latency, swimming speed and quadrant preference (Stoelting Co., Wood Dale, IL, USA).

### 2.3. Search Strategy Analysis 

During the acquisition training and probe trial, the search strategies were analyzed and classified using Pathfinder (Jason Snyder Lab, Vancouver, BC, Canada) [38] as previously described [27]. We adjusted the spatial parameters according to our experimental setup as follows: goal position [x/y]: 275, 775; goal diameter: 200 mm; maze diameter: 1100 mm; maze center [x/y]: 550, 550 mm; angular corridor width: 40°; chaining annulus width: 200 mm; thigmotaxis zone size: 50 mm.

**Figure 1:**
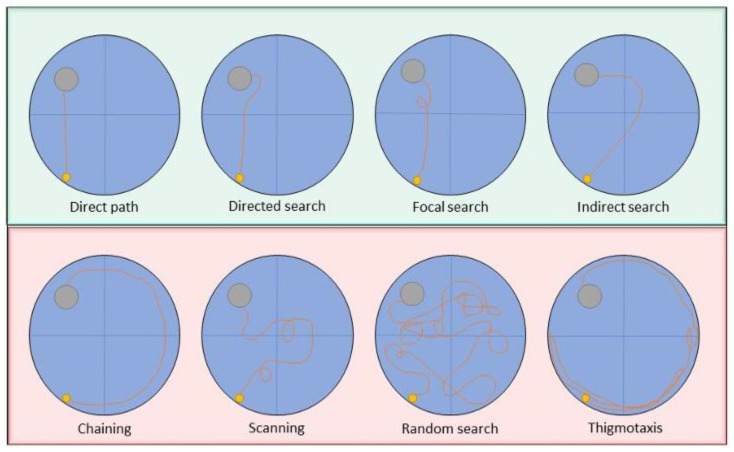
Search strategy classification by the Pathfinder system. Hippocampus-dependent strategies are also referred to as spatial strategies (green), whereas not-hippocampus-dependent are known as non-spatial strategies (red).

According to the Pathfinder system, search strategies can be classified into 8 different categories (Figure 1), which can further be divided into ‘spatial’ and ‘non-spatial’ strategies.

Spatial strategies include ‘direct path’, ‘directed search’, ‘directed search’, ‘focal search’ and ‘indirect search’. Thereby, a ‘direct path’ is used when the animal swims in an almost perfect path to the platform with minimal deviation (Ideal path error [IPE] ≤1250 mm; Heading error ≤40°). ‘Directed search’ is defined as a swimming strategy with little deviation from the direct path (time in angular corridor at least 70% of trial; distance covered ≤ 4000 mm; IPE ≤ 15,000 mm). ‘Focal search’ refers to a search in a limited space (distance to swim path centroid ≤ 30% of radius; distance to goal ≤ 30% of radius; distance covered ≥ 1000 mm and ≤ 4000 mm). ‘Indirect search’ describes a targeted spatial search with a major directional error (IPE ≤ 3000 mm; average heading error <360°).

In contrast, ‘chaining’, ‘scanning’, ‘random search’, and ‘thigmotaxis’ are considered as non-spatial search strategies. Thereby, an animal that swims in a constant distance from the wall uses a ‘chaining’ strategy to find the platform (time in annulus zone ≥ 90% of trial; quadrants visited = 4; area of maze traversed ≤ 40% of maze). ‘Scanning’ describes a random strategy in which the wall of the pool is avoided (area of maze traversed ≥ 5% and ≤20% of maze; average distance to maze center ≤60% of radius). A ‘Random search’ strategy does not feature any spatial search pattern (area of maze traversed ≥ 10% of maze). Furthermore, ‘thigmotaxis’ refers to a swimming pattern close to the wall of the pooll (time in full thigmotaxis zone ≥ 65% of trial; time in smaller thigmotaxis zone ≥ 35% of trial; total distance covered ≥ 4000 mm) [27,38].

### 2.4. Cognitive Score

To further assess the cognitive performance of mice, a cognitive score was calculated as a function of the search strategy used [39]. 

Spatial strategies corresponded to higher cognitive scores than non-spatial ones: direct path = 6; directed search = 5; focal search = 4; indirect search = 4; chaining = 3; scanning = 2; random search = 1; thigmotaxis = 0. For each mouse, the cognitive score was averaged per day and normalized to six, the highest possible score.

### 2.5. Statistical Analysis

For comparison of escape latency, swimming speed and cognitive score in the acquisition training we used two-way analysis of variance (ANOVA) followed by Bonferroni multiple comparisons. Time in the quadrant in the probe trial was analyzed using one-way analysis of variance (ANOVA) followed by Bonferroni multiple comparison. Differences between swimming speed and cognitive scores in the probe trial were tested with an unpaired *t*-test. For comparison of the search strategies between groups, a chi-square analysis was performed. 

Significance levels were defined as follows: *** *p* < 0.001, ** *p* < 0.01, * *p* < 0.05. All data were analyzed using GraphPad Prism 9.1.2 (GraphPad Software, San Diego, CA, USA).

## 3. Results

### 3.1. Age- and Sex-Dependent Spatial Learning Deficits of 5xFAD Mice in the Acquisition Training

The cued training showed that 5xFAD and WT mice, regardless of sex and age, had intact vision and the motor skills to perform the test as all mice displayed increasingly shorter escape latencies over the training phase. Twenty-four hours after the cued training, mice underwent an acquisition training to test their ability to find the location of a submerged platform using distal and proximal cues. 

**Figure 2:**
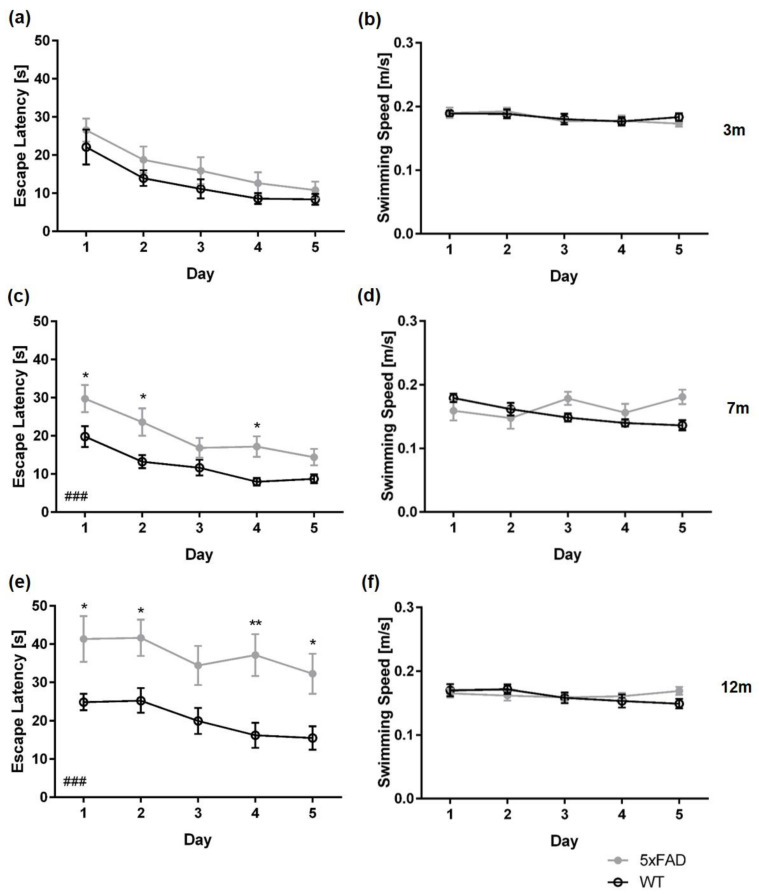
Female 5xFAD mice display age-dependent spatial learning deficits in the acquisition training. Escape latencies of 3- (**a**), 7- (**c**), and 12-month-old mice (**e**). Swimming speed did not differ between 5xFAD and WT mice at any age tested (**b**,**d**,**f**). Data presented as mean ± S.E.M., *n* = 10–13. Two-way repeated measures ANOVA: ### *p* < 0.001; Bonferroni: * *p* < 0.05, ** *p* < 0.01.

**Figure 3:**
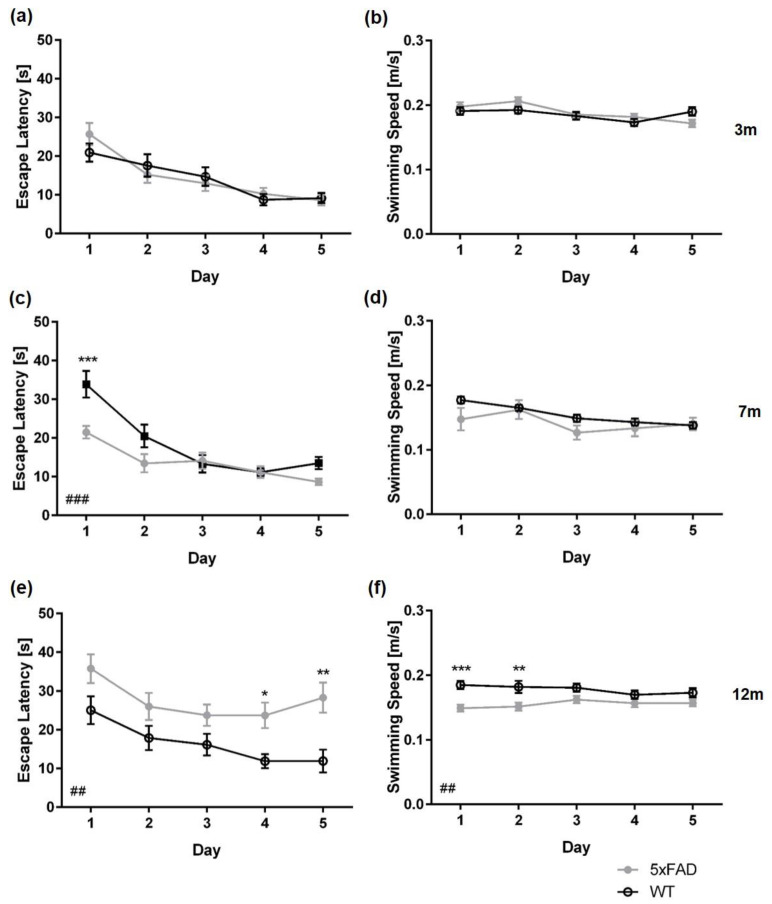
Male 5xFAD mice display age-dependent spatial learning deficits in the acquisition training. Escape latencies and swimming speed of 3- (**a**,**b**), 7- (**c**,**d**), and 12-month-old mice (**e**,**f**). Data presented as mean ± S.E.M., *n* = 10–13. Two-way repeated measures ANOVA: ## *p* < 0.01; ### *p* < 0.001; Bonferroni: * *p* < 0.05, ** *p* < 0.01, *** *p* < 0.001.

Between three-month-old 5xFAD and same-aged WT mice, the escape latency did not differ across the five days of acquisition training, regardless of sex (Figure 2a: *female* F(1,23) = 1.774, *p* = 0.1960; Figure 3a: *male* F(1,24) = 0.4099, *p* = 0.8413). In contrast, a significant main effect of genotype was found in female and male 7- and 12-month-old mice, with 5xFAD mice showing longer escape latencies than same-aged WT animals (Figure 2c,e: *female 7 m* F(1,24) = 17.18, *p* = 0.0004; *female 12 m* F(1,21) = 17.80, *p* = 0.0004; Figure 3c,e: *male 7 m* F(1,24) = 8.914, *p* = 0.0064; *male 12 m* F(1,21) = 9.091, *p* = 0.0066). 

Female seven-month-old WT mice performed better than 5xFAD animals on days one, two, and four of the acquisition training (Figure 2c: *day 1*, *day 2*, *day 4*: *p* < 0.05). In contrast, seven-month-old male WT mice performed significantly better than 5xFAD animals only on the first day of acquisition training. (Figure 3c: *day 1*: *p* < 0.001).

Furthermore, 12-month-old female 5xFAD mice required significantly more time to find the hidden platform on days 1, 2, 4, and 5 of the acquisition training than WT animals (Figure 2e: *all p* < 0.01). In addition, 12-month-old male WT mice performed significantly better than 5xFAD mice on days 4 and 5 of the acquisition training (Figure 3e: *day 4 p* < 0.05; *day 5 p* < 0.01). 

Furthermore, 12-month-old male 5xFAD mice performed significantly better than same-aged female 5xFAD mice (F(1,22) = 4.519, *p* = 0.045).

Swimming speed did not differ between female WT and 5xFAD mice at any age tested (Figure 2b,d,f: *3 m* F(1,23) = 0.06218, *p* = 0.8053; *7 m* F(1,24) = 1.465, *p* = 0.2379; *12 m* F(1,21) = 0.0930, *p* = 0.7634). In addition, swimming speed between three- and seven-month-old male WT and 5xFAD mice did not differ significantly (Figure 3b,d: *3 m* F(1,24) = 1.774, *p* = 0.1960; *7 m* F(1,24) = 2.213, *p* = 0.1480). In contrast, 12-month-old male 5xFAD mice swam significantly slower than WT animals (Figure 3f: *12 m* F(1,21) = 11.98, *p* = 0.0023; *day 1*, *p* < 0.001; *day 2*: *p* < 0.01).

In summary, male and female 5xFAD animals displayed age-dependent spatial learning deficits in the acquisition training.

### 3.2. Age- and Sex-Dependent Spatial Reference Memory Deficits in 5xFAD Mice in the Probe Trial

Twenty-four hours after the last day of acquisition training, a probe trial was conducted to assess spatial reference memory. During the 60 s of the probe trial, the percentage of the time spent swimming in each quadrant was recorded. 

**Figure 4:**
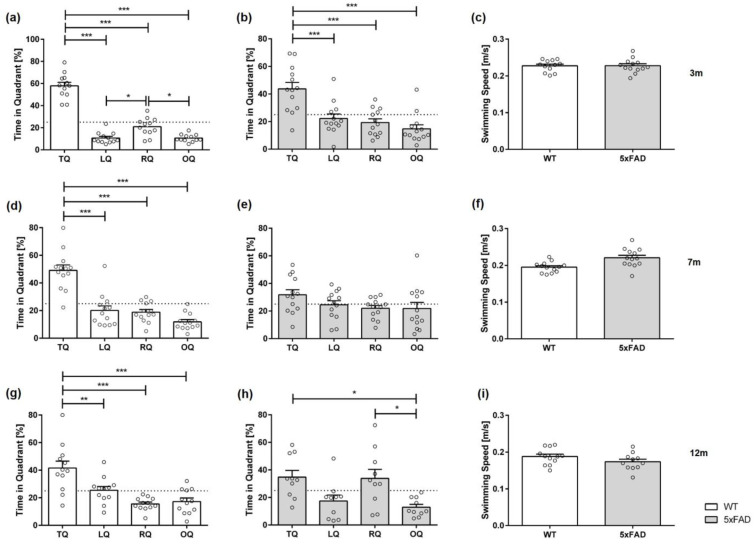
Female 5xFAD mice show age-dependent deficits in spatial reference memory in the probe trial. WT animals (**a**,**d**,**g**) and 3-month-old 5xFAD mice (**b**) showed a clear preference for the target quadrant during the probe trial. In contrast, 7- and 12-month-old 5xFAD mice demonstrated no clear preference for the target quadrant (**e**,**h**). Swimming speed did not vary between 5xFAD and WT mice (**c**,**f**,**i**). TQ = target quadrant, LQ = left quadrant, RQ = right quadrant, OQ = opposite quadrant. Data presented as mean ± S.E.M., *n* = 10–13. Quadrant preference: one-way repeated measures ANOVA followed by Bonferroni correction for multiple comparisons: * *p* < 0.05, ** *p* < 0.01, *** *p* < 0.001. Swimming speed: unpaired *t*-test.

**Figure 5:**
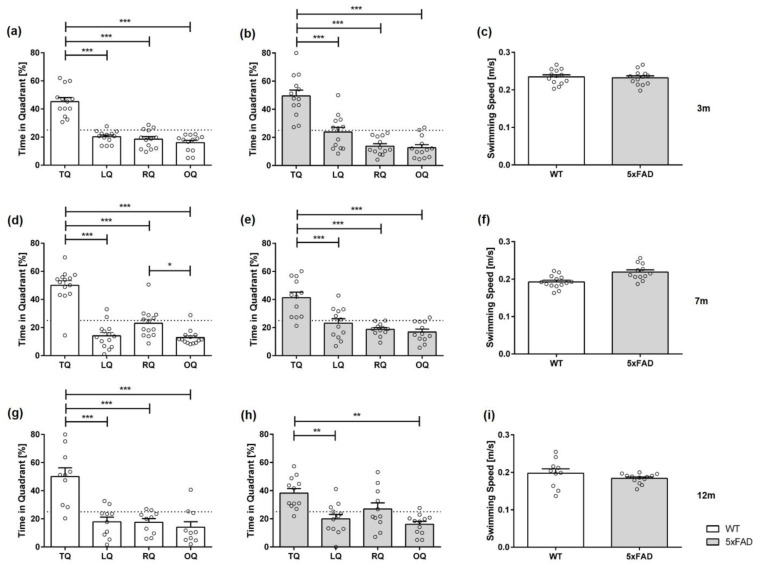
Aged male 5xFAD mice show mild spatial reference memory deficits in the probe trial. 3- and 7-month-old 5xFAD (**b**,**e**) mice and WT animals (**a**,**d**,**g**) showed a clear preference for the target quadrant. In contrast, 12-month-old 5xFAD mice demonstrated no clear preference for the target quadrant (**h**). No difference in swimming speed between 5xFAD and WT mice (**c**,**f**,**i**). TQ = target quadrant, LQ = left quadrant, RQ = right quadrant, OQ = opposite quadrant. Data presented as mean ± S.E.M., *n* = 10–13. Quadrant preference: one-way repeated measures ANOVA followed by Bonferroni correction for multiple comparisons: * *p* < 0.05, ** *p* < 0.01, *** *p* < 0.001. Swimming speed: unpaired *t*-test.

At three months of age, all animals, regardless of genotype and sex, showed a significant preference for the target quadrant (Figure 4a,b: *female 3 m WT* F(3,44) = 104.7, *p* < 0.001; *female 3 m 5xFAD* F(3,48) = 13.87, *p* < 0.001; Figure 5a,b: *male 3 m WT* F(3,48) = 47.09, *p* < 0.001; *male 3 m 5xFAD* F(3,48) = 32.23, *p* < 0.001).

In addition, seven-month-old female and male WT mice as well as male 5xFAD mice showed a clear preference for the target quadrant (Figure 4d: *female 7 m WT* F(3,48) = 32.51, *p* < 0.001; Figure 5d,e: *male 7 m WT* F(3,52) = 45.40, *p* < 0.001; *male 7 m 5xFAD* F(3,44) = 16.08, *p* < 0.001). In contrast, female 5xFAD mice demonstrated no preference for the target quadrant, indicating spatial reference memory deficits (Figure 4e: *female 7 m 5xFAD* F(3,48) = 1.906, *p* = 0.1412). 

Furthermore, 12-month-old WT mice, independent of sex, spent significantly more time in the target quadrant (Figure 4g: *female 12 m WT* F(3,44) = 13.47, *p* < 0.001; Figure 5g: *male 12 m WT* F(3,36) = 15.65, *p* < 0.001). However, 12-month-old female 5xFAD mice did not show a preference for the target quadrant (Figure 4h: F(3,40) = 2.978, *p* = 0.428; *LQ* vs. *RQ p* < 0.05). Similarly, 12-month-old male 5xFAD mice demonstrated no clear preference for the target quadrat (Figure 5h: F(3,48) = 7.247, *p* = 0.0004; *TQ* vs. *RQ p* < 0.05, *TQ* vs. *OQ p* < 0.01).

Swimming speed in the test trial did not differ between 5xFAD and WT mice in any age tested, regardless of gender (Figure 4c,f,i: *female 3 m* F(12,11) = 1.394, *p* = 0.9597; *female 7 m* F(12,12) = 3.050, *p* = 0.0810; *female 12 m* F(10,11) = 1.044, *p* = 0.1453; Figure 5c,f,i: *male 3 m* F(12,11) = 1.023, *p* = 0.7350; *male 7 m* F(11,13) = 1.147, *p* = 0.0560; *male 12 m* F(9,12) = 8.187, *p* = 0.2344).

In summary, the results of the acquisition and probe trial revealed age- and sex-dependent spatial learning and reference memory deficits in 5xFAD mice.

### 3.3. Search Strategy Analysis in Acquisition Training and Probe Trial

#### 3.3.1. Search Strategy Analysis of Female 5xFAD Mice

**Figure 6:**
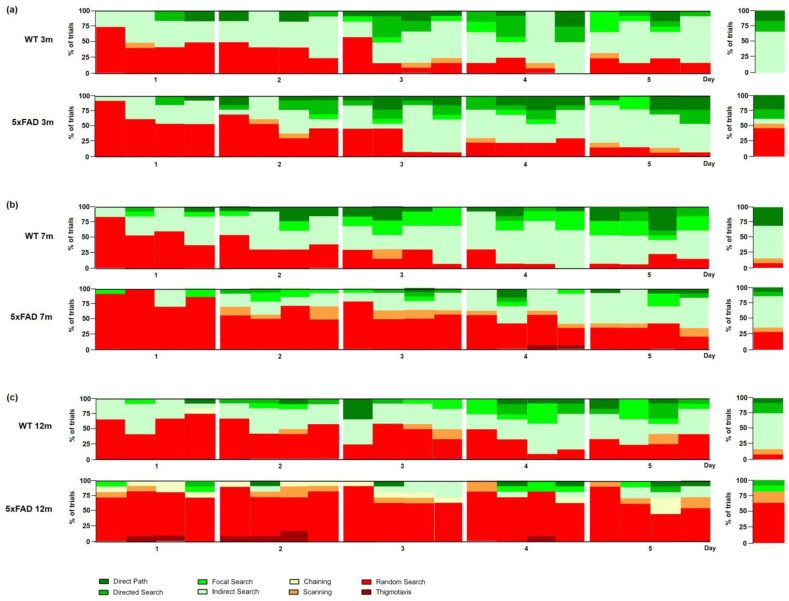
Qualitative analysis of spatial learning in female 5xFAD mice. Search strategies used by female 3 m- (**a**), 7 m- (**b**), and 12 m- (**c**) old 5xFAD and WT mice. Data represent the percentage of search strategies performed in each trial over the five days of acquisition training and during the probe trial. *n* = 10–13.

Search strategies between female three-month-old WT and 5xFAD mice did not differ significantly in the acquisition training (Figure 6a: *Day 1 p* = 0.5878, *Day 2 p* = 0.0558, *Day 3 p* = 0.7399, *Day 4 p* = 0.5661, *Day 5 p* = 0.4581). During the first day of acquisition training, 5xFAD and WT mice used primarily a ‘random search’ strategy (Table S1, *WT* 52%, *5xFAD* 66%). Over the training days, WT and 5xFAD mice used less non-spatial search strategies, and during the last day of acquisition training, both genotypes used predominantly an ‘indirect’ search strategy (*WT* 54%, *5xFAD* 56%).

**Figure 7:**
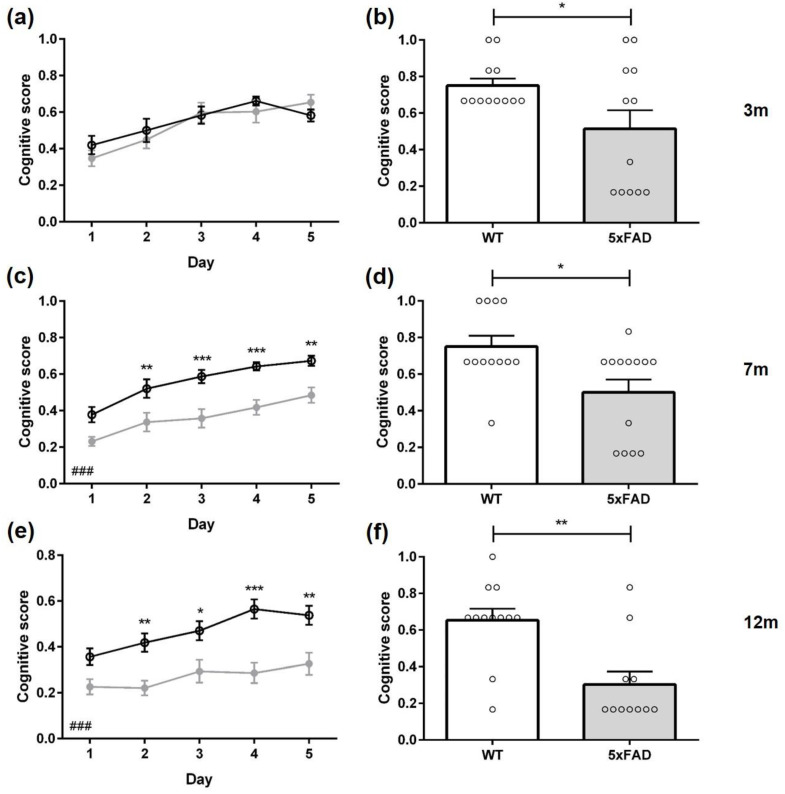
Cognitive scores of female 5xFAD mice. The cognitive scores of 5xFAD decreased age-dependently in the acquisition training (**a**,**c**,**e**) and the probe trial (**b**,**d**,**f**). Data presented as mean ± S.E.M., *n* = 10–13. Acquisition training: two-way repeated measures analysis of variance (ANOVA) followed by Bonferroni multiple comparisons. ANOVA: ### *p* < 0.001; Bonferroni: * *p* < 0.05, ** *p* < 0.01, *** *p* < 0.001. Probe trial: unpaired *t*-test: * *p* < 0.05, ** *p* < 0.01.

The cognitive level of different swimming strategies can be quantified using a cognitive score that evaluates swimming strategies according to their relevance to spatial learning. A higher cognitive score indicates that mice use primarily spatial learning strategies, while mice with a lower cognitive score rely mainly on non-spatial learning strategies [27]. The cognitive score of female three-month-old 5xFAD mice did not differ significantly from same-aged WT mice in the acquisition training (Figure 7a: F(1,23) = 0.1596, *p* = 0.6932).

During the probe trial, 53% of 3-month-old 5xFAD animals used non-spatial search strategies with predominately ‘random search’. In contrast, WT mice relied on different forms of spatial search strategies (Figure 6a: *p* = 0.0133). In addition, female three-month-old 5xFAD mice showed a lower cognitive score than WT animals (Figure 7b: F(12,11) = 7.002, *p* = 0.0404).

Female seven-month-old 5xFAD and WT mice used mainly ‘random search’ strategies (Table S1, *WT* 60%, *5xFAD* 88%) during the first day of acquisition training. Over the subsequent days, the fraction of ‘random search’ strategies used by WT animals declined and by day four WT mice used mainly spatial strategies. In contrast, female 5xFAD mice did not improve their swimming strategies as quickly (*Day 2, 3, 4 p* < 0.001, *Day 5 p* = 0.0016), and still employed non-spatial strategies (‘random search’ and ‘scanning’) in 40% of the trials on the last day of acquisition training. In addition, 5xFAD mice showed a lower cognitive score on almost all training days (Figure 7c: F(1,25) = 37.80, *p* < 0.001). 

During the probe trial, 36% of 7-month-old 5xFAD mice used non-spatial search strategies, whereas only 15% of WT animals relied on different forms of non-spatial search strategies. Furthermore, seven-month-old 5xFAD mice displayed a lower cognitive score than same-aged WT animals (Figure 7d: F(13,12) = 1.510, *p* = 0.0134).

During the first day of acquisition training, female 12-month-old 5xFAD and WT animals used mainly a ‘random search’ strategy to locate the platform (Table S1, *WT* 63%, *5xFAD* 73%). Over the training days, WT mice used less non-spatial strategies, especially ‘random searches’. In contrast, 5xFAD mice used predominately non-spatial strategies on all five training days (*Day 2* 95%, *Day 3* 85%, *Day 4* 82%, *Day 5* 81%). 

The search strategies used by 12-month-old female 5xFAD animals differed significantly from WT animals on all days of acquisition training (Figure 6c: *Day 1 p* = 0.0012, *Day 2 p* < 0.001, *Day 3 p* = 0.0076, *Day 4 p* < 0.001, *Day 5 p* = 0.0023). Overall, 5xFAD mice relied significantly more on non-spatial strategies than WT animals, and the cognitive score was significantly lower in 5xFAD mice in the acquisition training (Figure 7e: F(1,21) = 27.43, *p* < 0.001).

During the probe trial, 82% of female 12-month-old 5xFAD mice used non-spatial search strategies. In contrast, 83% of WT animals used spatial search strategies with predominantly ‘indirect search’ (*p* = 0.0018). Furthermore, 5xFAD animals demonstrated a significantly lower cognitive score than same-aged WT mice (Figure 7f: F(10,11) = 1.141, *p* = 0.0013).

Between 3-, 7-, and 12-month-old WT mice, the cognitive score did not differ (F(2,34) = 4.478, *p* = 0.4602). However, 5xFAD mice showed a decline in cognitive scores with increasing age (F(2,35) = 16.56, *p* < 0.001).

#### 3.3.2. Search Strategy Analysis of Male 5xFAD Mice

**Figure 8:**
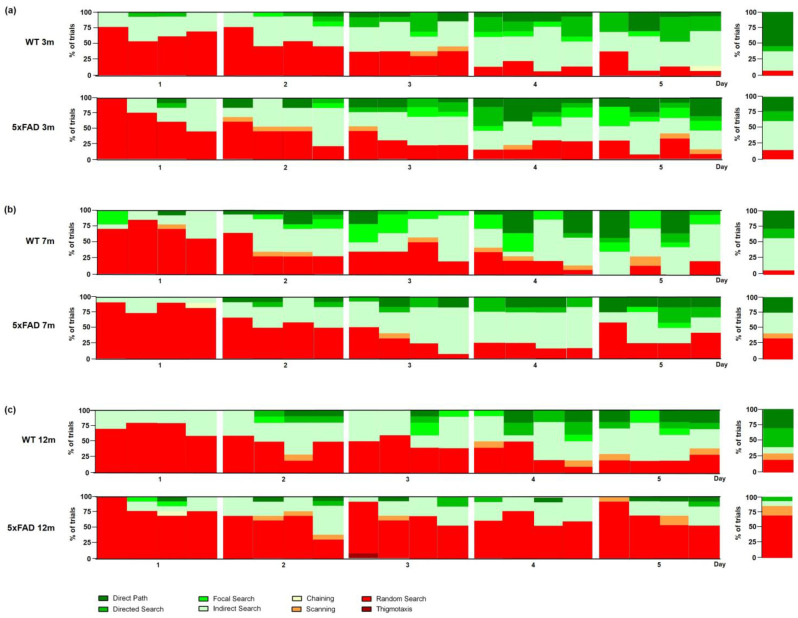
Qualitative analysis of spatial learning in male 5xFAD mice. Search strategies used by male 3 m- (**a**), 7 m- (**b**), and 12 m- (**c**) old 5xFAD and same-aged WT mice. Data represent the percentage of search strategies performed in each trial over the 5 days of acquisition training and during the probe trial. *n* = 10–13.

**Figure 9:**
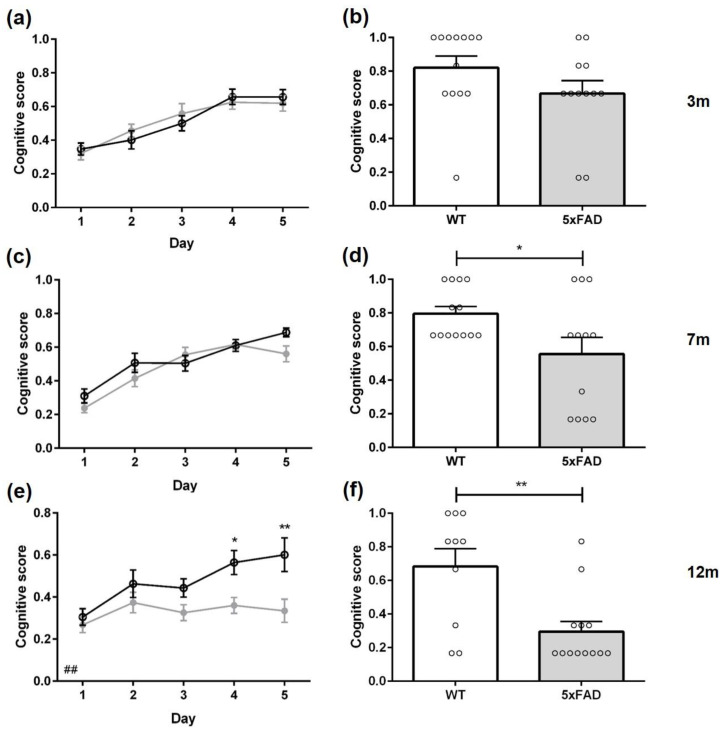
Cognitive scores of male 5xFAD mice. The cognitive scores of 5xFAD mice decreased age-dependently in the acquisition training (**a**,**c**,**e**) and the probe trial (**b**,**d**,**f**). Data presented as mean ± S.E.M., *n* = 10–13. Acquisition training: two-way repeated measures analysis of variance (ANOVA) followed by Bonferroni multiple comparisons. ANOVA: ## *p* < 0.01; Bonferroni: * *p* < 0.05, ** *p* < 0.01. Probe trial: unpaired *t*-test: * *p* < 0.05, ** *p* < 0.01.

Male three-month-old WT and 5xFAD mice showed comparable search strategies over the acquisition training (Figure 8a: *Day 1 p* = 0.6214, *Day 2 p* = 0.2813, *Day 3 p* = 0.8657, *Day 4 p* = 0.3573). Furthermore, 5xFAD mice did not show a different cognitive score than WT mice in the acquisition training (Figure 9a: F(1,24) = 0.9451, *p* = 0.9234). 

During the probe trial, the search strategies did not differ between 5xFAD and WT animals (Figure 8a: *p* = 0.4459). In addition, 5xFAD mice exhibited a similar cognitive score compared with WT animals (Figure 9b: F(11,12) = 1.135, *p* = 0.1492). 

Male seven-month-old 5xFAD and WT animals used predominantly a ‘random search’ strategy on the first day of acquisition training (Figure 8b: *Day 1 p* = 0.2275; Table S2). Over the training days, non-spatial search strategies decreased in both groups. However, search strategies differed significantly between male WT and 5xFAD mice on days three, four, and five of the acquisition training (*Day 3 p* = 0.0155, *Day 4 p* = 0.0010, *Day 5 p* = 0.0051). On the last day of acquisition training, WT mice used nearly no non-spatial strategies, while male 5xFAD mice still relied on a ‘random search’ strategy in 38% of the trials. However, the cognitive score did not differ between 5xFAD and WT animals in the acquisition training (Figure 9c: F(1,24) = 1.634, *p* = 0.2134).

During the probe trial, WT animals used significantly more spatial search strategies than 5xFAD mice (Figure 8b: *p* = 0.0373). While 93% of WT mice used spatial search strategies with predominantly ‘indirect search’ and ‘direct path’, 5xFAD mice used a mixture of non-spatial and spatial strategies. In addition, seven-month-old male 5xFAD mice showed a significantly lower cognitive score than WT animals (Figure 9d: F(11,12) = 4.948, *p* = 0.0323).

During the first day of acquisition training, male 12-month-old 5xFAD and WT animals used mainly a ‘random search’ strategy (Table S2, *WT* 73%, *5xFAD* 81%) to locate the hidden platform. During the acquisition training, WT mice used fewer ‘random searches’. In contrast, male 5xFAD mice used non-spatial strategies with predominantly ‘random search’ on all days (*Day 2* 66%, *Day 3* 69%, *Day 4* 64%, *Day 5* 73%). The overall search strategies used by 12-month-old male 5xFAD animals differed significantly from WT animals on the last 2 days of acquisition training (Figure 8c: *Day 1 p* = 0.1716, *Day 2 p* = 0.474; *Day 3 p* = 0.1607, *Day 4 p* = 0.0027, *Day 5 p* < 0.001). In addition, male 12-month-old 5xFAD mice showed a decreased cognitive score in the acquisition training (Figure 9e, F(1,21) = 10.46, *p* = 0.004).

During the probe trial, 84% of 12-month-old 5xFAD mice used non-spatial search strategies, while only 36% of WT animals used non-spatial search strategies (Figure 8c: *p* = 0.015). Furthermore, male 5xFAD animals showed a significantly lower cognitive score than same-aged WT mice (Figure 9f: F(9,12) = 2.425, *p* = 0.0030).

The cognitive score did not differ between 3-, 7-, and 12-month-old male WT mice (F(2,34) = 0.7807, *p* = 0.5178). However, the cognitive score of male 5xFAD mice decreased with age in the acquisition training (F(2,35) = 13.35, *p* < 0.001).

## 4. Discussion

Spatial reference memory is one of the first mechanisms to be impaired in the progression of AD and there is clear evidence that women are more affected than men [40]. The cognitive deficits in 5xFAD mice have been extensively studied using the MWM and other tests, but most of the previous studies do not classify the search strategy and only a few compare by sex [31,41,42,43]. In the present study, a classification of the swimming search pattern in addition to the conventional MWM paradigm provided a broader picture of the learning and navigation impairments in male and female mice. Similar to humans, we observed that these deficits are age- and sex-dependent in 5xFAD mice.

The MWM is routinely used for analyzing memory deficits in AD mice, including the 5xFAD model. Most studies have used conventional behavior measures based on average performance, such as escape latencies [31,44,45,46,47,48]. In line with previous studies [31,45,49], we could demonstrate age- and sex-dependent deficits in spatial learning and reference memory by using conventional behavior analysis of the escape latencies and quadrant preference. The majority of previous studies reported learning impairments between 4 and 10 months of age [31,45,49,50,51,52,53]. Our findings show first spatial learning deficits in 7-month-old female 5xFAD and 12-month-old male 5xFAD mice, respectively. The different onset of memory deficits in different studies are likely due to the testing procedure and apparatus design.

Evaluating spatial working memory based on escape latencies provides an overall picture of memory performance but does not explain how an animal solves a spatial task. Therefore, a detailed analysis of the swimming strategies was performed for a better understanding of the behavioral differences between 5xFAD and WT mice. Overall, 5xFAD mice used quantitatively and qualitatively different search strategies than WT animals.

Detailed search strategy analyses have been performed in several AD mouse models including APP/PS1 [54,55], Tg4-42 [27], PDAPP [56], and TgCRND8 [57,58]. However, there are only a few studies that have briefly addressed the classification of search strategies in 5xFAD mice [59,60]. To our knowledge, the current study is the first detailed analysis of the performance of 5xFAD mice in the MWM that includes search strategies.

Learning in the MWM forces mice to develop efficient navigation strategies that focus on local landmarks to find the hidden platform [19]. Consistent with previous studies, WT animals, regardless of age or sex, used more spatial strategies the better they mastered the MWM task [27,55,61]. In contrast, 5xFAD mice showed a strong reliance on non-spatial strategies, especially random search, with increasing age. In line with these findings, Sin and colleagues (2020) demonstrated an increased use of non-spatial strategies in four-month-old 5xFAD mice compared to WT animals in the acquisition training [60]. In addition, Cho et al. (2014) showed that six-month-old 5xFAD mice used different strategies to locate the platform in the MWM. However, they only distinguished between ‘cued strategy’ and ‘place/spatial strategy’. Interestingly, the strategy preference depended on the sequence of place/spatial and cued training [59].

Similar to WT animals, female three-month-old and male three- and seven-month-old 5xFAD mice adapted their search strategies during the acquisition training and used mainly spatial strategies towards the end. These changes were probably the primary reason for the improved performance over the training days.

Strikingly, analysis of the search strategies in the probe trial revealed early behavior changes in female three-month-old 5xFAD mice that would have been overlooked when focusing only on quadrant preferences. While young 5xFAD mice demonstrated a clear preference for the target quadrant, more than half of female 5xFAD animals used non-spatial search strategies to solve the task. In contrast, none of the same-aged control WT animals used a non-spatial search strategy in the probe trial. Similarly, while seven-month-old male 5xFAD animals showed a preference for the target quadrant, they used significantly more non-spatial search strategies than same-aged control animals. These deficits did not result in reference memory deficits detected by the classical MWM analysis because the non-spatial search strategies employed by 5xFAD mice were sufficient to find the right quadrant. However, detailed analysis of the swimming strategies detected allocentric memory deficits in the probe trial of these mice. 

12-month-old 5xFAD mice, regardless of sex, showed spatial learning memory and reference memory deficits. Both sexes used significantly more non-spatial search strategies than their age- and sex-matched controls during the acquisition training and the probe trial. The predominant use of ‘random search’ strategies in aged 5xFAD mice resulted in almost no improvement in their escape latency over the acquisition training. Similar behavior has been described in Tg4-42 transgenic mice, with 7- and 12-month-old animals displaying spatial learning deficits while relying mostly on non-spatial search strategies [27]. Moreover, working memory deficits in six-month-old TgCRND8 mice were associated with mostly non-spatial chaining strategies [57]. Similarly, Brody and Holtzman (2005) showed that deficits in PDAPP mice in the MWM are associated with the use of primary non-spatial strategies and, in particular, repetitive looping strategies [56].

The 5xFAD model develops a number of AD-related neuropathologies that likely contribute to the observed age-related memory deficits. 5xFAD mice display an early plaque formation and intraneuronal Aβ aggregation [28,34,62]. Plaque pathology in the hippocampus, a brain region critical for spatial memory, is most pronounced in the subiculum and associated with neuron loss at nine months [28,63]. In addition, impaired basal synaptic transmission as well as reduced long-term potentiation are observed in the CA1 in six-month-old animals [31,64,65]. Furthermore, reduced adult neurogenesis in the dentate gyrus occurs as early as two months [66,67]. Andersen et al. (2021) described dysfunctional excitatory neuronal signaling, impaired cellular metabolism, and alterations in the proteome in the hippocampus of young 5xFAD mice [68]. In addition, increased astro- and microgliois are described in the hippocampus after two months of age [33,69]. However, further research is needed to establish how these pathologies contribute to the learning and memory deficits in 5xFAD mice.

It is important to note that the observed deficits in spatial working memory in aged 5xFAD mice cannot be attributed to increased thigmotaxis. Thigmotaxis, a behavior often described in transgenic mice, is associated with swimming along the pool wall and is often accompanied by longer escape latencies [70,71,72]. As a result, spatial learning ability cannot be appropriately assessed. However, 5xFAD mice did not differ in thigmotaxis swims or floating rate at any age tested. 

Moreover, the observed memory deficits are likely not due to the previously described motor impairments in 5xFAD mice [30,34,73]. Motor impairments, here measured by swimming speed, can possibly interfere with cognitive behavior readouts [55,74]. O’Leary et al. (2020) detected first motor impairments in 9–10 months old 5xFAD mice and a reduced locomotor activity and impaired balance after 12 months [30,73]. Similarly, Jawhar and colleagues described motor impairments in female 5xFAD mice beginning at nine months of age [34]. O’Leary and Brown (2022) described decreased swimming speed in male and female 12- and 15-month-old 5xFAD animals. In contrast, we only detected a slightly altered swimming speed in 12-month-old male 5xFAD mice, as they swam significantly slower on the first two days of acquisition training. Is seems that the reduced swimming speed observed here in aged male 5xFAD mice is due to the unfamiliar task rather than the animal’s inability to swim. Importantly, the slower swimming speed observed in the current study did not result in lower escape latencies. 

Women are disproportionately affected by AD in terms of both severity and prevalence [75,76,77]. After the first diagnosis of mild cognitive impairment, women show a faster cognitive decline than men as well as different cognitive and psychiatric symptoms [77]. However, the reason for this discrepancy is still not yet known. In line with the observations in patients, female 5xFAD mice showed earlier spatial learning deficits as well as a faster disease progression.

Several studies showed a more severe or earlier amyloid pathology, including Aβ plaques and elevated levels of APP and Aβ in female 5xFAD mice [28,78,79,80,81]. Interestingly, Bundy et al. (2019) demonstrated higher levels of human APP and PS1 mRNA expression in the hippocampus of female four-month-old 5xFAD mice compared to males. Furthermore, a gene ontology study revealed a sex-specific molecular pattern especially in genes associated with the immune system [82]. In addition, several studies described sex-specific behavioral differences in 5xFAD mice, ranging from differences in motor skills and odor recognition to deficits in working memory [31,73,83,84]. Similar to our results, O’Leary and Brown (2022) showed sex- and age-dependent deficits in the MWM with female 5xFAD mice performing worse and showing more severe reversal learning impairments. 

To our knowledge, the current study is the first to show age- and sex-specific differences in 5xFAD or other transgenic AD models with respect to swimming strategies in the MWM, with female 5xFAD mice developing earlier deficits in spatial navigation. In contrast, in young APP/PS1 animals, search strategy alterations in the MWM were only detected in male animals [55]. Interestingly, gender differences in navigation strategy and efficiency were also described in healthy humans [85]. Furthermore, a robust gender difference in the effectiveness and utility of the virtual Morris water task could be detected between men and women [86]. Several authors suggest that the observed sex differences in humans may be dependent on sex hormones. The hippocampus is quite sensitive to estrogen peaks, so the lack of it and the hormonal fluctuations may more greatly predispose the female hippocampus to neurodegeneration [87]. Importantly, even adult healthy women have been described to use more egocentric strategies for spatial navigation [85].

We acknowledge that this work has some limitations. While the current study extends previous MWM studies by including search strategies in addition to escape latencies, it must be noted that Pathfinder can only identify which search strategy is predominantly used but does not detect transitions between different strategies during a single trial. However, Pathfinder, unlike a human observer, allows complete objectivity in the classification of individual search strategies [38]. Furthermore, we did not investigate a possible molecular mechanism that could explain the altered search strategies. Nevertheless, the swimming strategies give more insight into the observed memory deficits of 5xFAD mice in the MWM and reveal early behavior changes. 

In summary, our detailed longitudinal analysis revealed age- and sex-dependent differences in the pattern of search strategies employed by 5xFAD mice. The 5xFAD model is commonly used in preclinical studies to evaluate possible treatment strategies for AD. Therefore, analyzing swim strategies next to the classical analysis of the MWM may provide advantages in detecting early and subtle changes, thus helping to develop better-informed treatment strategies in the future.

## Data Availability

The data included in this study are available upon request.

## Supplementary Materials

The following supporting information can be downloaded at: https://www.mdpi.com/article/10.3390/biomedicines11020599/s1, Table S1: Percentage of search strategies used during the acquisition training of female mice; Table S2: Percentage of search strategies used during the acquisition training of male mice.
