# Supplementary material for: Search Strategy Analysis of 5xFAD Alzheimer Mice in the Morris Water Maze Reveals Sex- and Age-Specific Spatial Navigation Deficits
Source: Biomedicines. 2023 Feb 17;11(2):599. doi: 10.3390/biomedicines11020599 (PMC9953202; doi:10.3390/biomedicines11020599)
Supplement: Supplementary file 1 [file biomedicines-11-00599-s001.zip › biomedicines-2142014-supplementary.pdf]

Supplementary Materials

Search Strategy Analysis of 5xFAD Alzheimer Mice in the Morris Water Maze Reveals Sex- and Age-Specific Spatial Navigation Deficits

Carolina Quintanilla Sánchez, Franziska W. Schmitt, Nadine Curdt, Anna Celine Westhoff, Irina Wanda Helene Bänfer, Thomas A. Bayer and Yvonne Bouter

Table S1. Percentage of search strategies used during the acquisition training of female mice.

Strategy	Day 1		Day 2		Day 3		Day 4		Day 5	
3m female	**WT**	**5XFAD**	**WT**	**5XFAD**	**WT**	**5XFAD**	**WT**	**5XFAD**	**WT**	**5XFAD**
Direct Path	4.17	1.92	8.33	7.69	10.42	13.46	8.33	13.46	4.17	13.46
Directed Search	4.17	3.85	2.10	11.54	12.50	11.54	18.75	13.46	6.25	7.69
Focal Search	0.00	0.00	0.00	1.92	6.25	3.85	6.25	3.85	12.50	7.69
Indirect Search	37.5	28.85	50.00	25.00	41.67	44.23	52.08	42.31	54.17	55.77
Chaining	0.00	0.00	0.00	0.00	0.00	0.00	0.00	0.00	0.00	0.00
Scanning	2.10	0.00	0.00	3.85	4.17	0.00	2.08	1.92	2.08	3.85
Random Search	52.10	65.38	39.58	50.00	25	26.92	12.5	25.00	20.83	11.54
Thigmotaxis	0.00	0.00	0.00	0.00	0.00	0.00	0.00	0.00	0.00	0.00
7m female	**WT**	**5XFAD**	**WT**	**5XFAD**	**WT**	**5XFAD**	**WT**	**5XFAD**	**WT**	**5XFAD**
Direct Path	1.92	0.00	13.46	0.00	11.54	1.79	7.69	3.57	17.31	5.36
Directed Search	1.92	0.00	0.00	3.57	0.00	5.36	3.85	1.79	9.62	1.79
Focal Search	3.85	3.13	5.77	8.93	23.08	3.57	15.38	3.57	19.23	5.36
Indirect Search	32.69	9.38	42.31	19.64	40.38	21.43	61.54	37.50	40.38	46.43
Chaining	0.00	0.00	0.00	0.00	0.00	0.00	0.00	0.00	0.00	0.00
Scanning	0.00	0.00	0.00	10.71	3.85	8.93	0.00	5.36	0.00	7.14
Random Search	59.62	87.50	38.46	57.14	21.15	58.93	11.54	44.64	13.46	33.93
Thigmotaxis	0.00	0.00	0.00	0.00	0.00	0.00	0.00	3.57	0.00	0.00
12m female	**WT**	**5XFAD**	**WT**	**5XFAD**	**WT**	**5XFAD**	**WT**	**5XFAD**	**WT**	**5XFAD**
Direct Path	2.08	0.00	2.08	2.27	8.33	2.27	6.25	4.55	6.25	4.55
Directed Search	2.08	2.27	6.25	0.00	2.08	0.00	8.33	0.00	10.42	2.27
Focal Search	0.00	4.55	4.17	0.00	6.25	0.00	16.67	9.09	12.50	2.27
Indirect Search	31.25	0.00	33.33	2.27	35.42	13.64	41.67	4.55	35.42	11.36
Chaining	2.08	11.36	0.00	6.82	0.00	9.09	0.00	2.27	0.00	6.82
Scanning	0.00	4.55	2.08	9.09	6.25	4.55	0.00	4.55	4.17	9.09
Random Search	62.50	72.73	52.08	70.45	41.67	70.45	27.08	72.73	31.25	63.64
Thigmotaxis	0.00	4.55	0.00	9.09	0.00	0.00	0.00	2.27	0.00	0.00

Table S2. Percentage of search strategies used during the acquisition training of male mice.

Strategy	Day 1		Day 2		Day 3		Day 4		Day 5	
3m male	**WT**	**5XFAD**	**WT**	**5XFAD**	**WT**	**5XFAD**	**WT**	**5XFAD**	**WT**	**5XFAD**
Direct Path	0.00	1.92	1.92	7.69	7.69	9.62	11.54	21.15	11.54	19.23
Directed Search	3.85	1.92	3.85	1.92	13.46	11.54	17.31	11.54	23.08	5.77
Focal Search	0.00	0.00	3.85	1.92	1.92	5.77	7.69	9.62	1.92	17.31
Indirect Search	30.77	25.00	34.62	38.46	36.54	40.38	48.08	32.69	44.23	32.69
Chaining	0.00	0.00	0.00	0.00	0.00	0.00	0.00	0.00	1.92	0.00
Scanning	0.00	0.00	0.00	5.77	3.85	1.92	0.00	1.92	0.00	3.85
Random Search	65.38	71.15	55.77	44.23	36.54	30.77	15.38	23.08	17.31	19.23
Thigmotaxis	0.00	0.00	0.00	0.00	0.00	0.00	0.00	0.00	0.00	0.00
7m male	**WT**	**5XFAD**	**WT**	**5XFAD**	**WT**	**5XFAD**	**WT**	**5XFAD**	**WT**	**5XFAD**
Direct Path	1.79	0.00	10.71	6.25	5.36	8.33	19.64	8.33	19.64	18.75
Directed Search	0.00	0.00	1.79	4.17	1.79	8.33	1.79	14.58	7.14	10.42
Focal Search	5.36	0.00	7.14	0.00	19.64	0.00	16.07	0.00	16.07	4.17
Indirect Search	19.64	12.50	39.29	33.33	35.71	52.08	35.71	56.25	44.64	29.17
Chaining	0.00	2.08	0.00	0.00	0.00	0.00	0.00	0.00	0.00	0.00
Scanning	1.79	0.00	3.57	0.00	1.79	2.08	5.36	0.00	3.57	0.00
Random Search	71.43	85.42	37.50	56.25	35.71	29.17	21.43	20.83	8.93	37.50

Thigmotaxis	0.00	0.00	0.00	0.00	0.00	0.00	0.00	0.00	0.00	0.00

12m male	**WT**	**5XFAD**	**WT**	**5XFAD**	**WT**	**5XFAD**	**WT**	**5XFAD**	**WT**	**5XFAD**
Direct Path	0.00	1.92	5.00	3.85	2.50	1.92	10.00	1.92	15.00	5.77
Directed Search	0.00	0.00	7.50	1.92	2.50	3.85	15.00	1.92	7.50	1.92
Focal Search	0.00	3.85	2.50	0.00	7.50	0.00	5.00	0.00	7.50	0.00
Indirect Search	27.50	11.54	37.50	30.77	40.00	23.08	35.00	32.69	42.50	19.23
Chaining	0.00	1.92	0.00	0.00	0.00	0.00	0.00	0.00	0.00	0.00
Scanning	0.00	0.00	2.50	5.77	0.00	1.92	5.00	0.00	5.00	5.77
Random Search	72.50	80.77	45.00	57.69	47.50	67.31	30.00	63.46	22.50	67.31
Thigmotaxis	0.00	0.00	0.00	0.00	0.00	1.92	0.00	0.00	0.00	0.00
